# Predictors of Major Lower Extremity Amputation in Type 2 Diabetic Patients With Diabetic Foot Ulcers: A Cross-Sectional Analytical Study

**DOI:** 10.7759/cureus.77339

**Published:** 2025-01-12

**Authors:** Neeraj Sharma, Cherring Tandup, Ashu Rastogi, Swapnesh Sahu, Arunanshu Behera, Ajay Savlania, Satish Subbiah Nagaraj, Basil Babu, Naveen Pentakota, Rahul Gupta

**Affiliations:** 1 General Surgery, Postgraduate Institute of Medical Education and Research, Chandigarh, IND; 2 Endocrinology and Diabetes, Postgraduate Institute of Medical Education and Research, Chandigarh, IND; 3 General Surgery, Ivy Hospital, Chandigarh, IND; 4 Public Health, Public Health Foundation of India, Himachal Pradesh, IND

**Keywords:** diabetic foot ulcers, inflammatory markers, lower extremity amputation, peripheral pulsation, predictive factors, wagner's grade

## Abstract

Introduction

Diabetic foot ulcers (DFUs) are a significant complication of diabetes mellitus, often leading to major lower extremity amputation. Identifying predictive factors for amputation can guide interventions to prevent this severe outcome. This study aimed to identify predictors of major lower extremity amputation in type 2 diabetic patients with DFUs.

Methodology

A cross-sectional analytical study was conducted at the Department of General Surgery, Postgraduate Institute of Medical Education and Research, Chandigarh, India, involving 101 patients with DFUs. The study assessed the association between various clinical and laboratory predictors and the necessity for major lower extremity amputation.

Results

Significant predictors for amputation included Wagner's grade ≥ 5, absent peripheral pulsation, clinical evidence of infection, and elevated levels of erythrocyte sedimentation rate and C-reactive protein.

Conclusion

This study highlights the importance of early identification of high-risk patients through detailed clinical assessment and laboratory investigations and underscores the need for aggressive management strategies targeting identified predictors to reduce the risk of major amputation among patients with DFUs.

## Introduction

Diabetes mellitus is a metabolic disorder characterized by glucose dysregulation. Among individuals with diabetes, approximately 90% to 95% have type 2 diabetes mellitus, while the remaining 5% to 10% have type 1 diabetes mellitus. The diagnosis of diabetes mellitus is typically based on fasting blood sugar levels or hemoglobin A1C (HbA1C) measurements [1]. Poorly controlled blood sugar levels significantly increase the risk of complications, including nephropathy, retinopathy, and neuropathy.

In patients with diabetic neuropathy, the loss of protective sensation and proprioception increases the likelihood of skin breakdown, particularly over the toes and feet [2]. These factors contribute to the development of diabetic foot syndrome (DFS), which the World Health Organization (WHO) defines as “ulceration of the foot (distally from the ankle, including the ankle) associated with neuropathy and various degrees of ischemia and infection” [3]. Wounds below the ankle in individuals with diabetes are referred to as diabetic foot ulcers (DFUs).

DFUs are a leading cause of hospitalization among individuals with diabetes and represent one of the most common and severe complications of the condition. These ulcers impose significant medical, social, and financial burdens on patients, their families, and society at large [4]. Approximately 40% to 60% of non-traumatic lower extremity amputations (LEAs) globally are attributed to diabetes-related complications, with 80% of these amputations preceded by DFUs [5].

Past studies have demonstrated that diabetes-related amputations - both major and minor - are associated with high mortality rates. The five-year survival rate following such amputations is alarmingly low, ranging from 41% to 48%. Even for patients undergoing minor amputations, the five-year survival rate is only 59% [6].

Understanding the risk factors for amputation is critical for individuals newly diagnosed with DFUs. By identifying these predictors, healthcare providers can implement targeted interventions to delay or prevent severe complications. In light of these considerations, we conducted a study aimed at identifying the potential risk factors for LEAs in patients with DFUs.

## Materials and methods

This cross-sectional analytical study was designed to identify predictors of major LEA among type 2 diabetic patients presenting with DFUs. Conducted at the Department of General Surgery, Postgraduate Institute of Medical Education and Research (PGIMER), Chandigarh, India, the study spanned over one year from January 1, 2022, to June 30, 2023. The study population comprised patients diagnosed with DFS through clinical examination, corroborated by adjunctive investigations. Eligibility was determined based on the following criteria: adults over 18 years with type 2 diabetes mellitus presenting with DFUs. Exclusions were applied to those unwilling to participate, those refusing consent for major LEA, previous recipients of endovascular interventions or grafting in the affected extremity, and patients with cancers or tumors of the lower extremity or who were pregnant.

Sample size calculation was based on a prevalence rate of 7.1% for major lower limb amputation among DFU patients, as reported by Perng et al., using a formula that accounted for a 95% confidence level and a precision level of 0.05, resulting in a final sample size of 101 patients [7].

The methodology encompassed detailed history taking, comprehensive clinical examination (including general physical and systemic examinations, diabetic foot examination according to Wagner’s grading, and assessment of chronic limb-threatening ischemia as per Rutherford’s classification), measurement of ankle brachial index (ABI) to screen for peripheral arterial disease, and further evaluation with USG Doppler and CT angiography as necessary. Laboratory investigations included complete hemograms, renal and liver function tests, lipid profile, and markers of infection such as erythrocyte sedimentation rate (ESR) and C-reactive protein (CRP), along with glycemic control assessment through HbA1C levels.

Patients were categorized based on the necessity of major LEA versus other treatments (debridement, minor amputation, revascularization surgery, or conservative management with antibiotics and/or antiseptic dressing). Major amputations included below-knee (transtibial) and above-knee (transfemoral) amputations, with decisions made by a general surgeon based on clinical findings, Wagner’s and Rutherford’s grading, tissue viability, and vascular supply assessment. Intra-operative findings and postoperative complications were noted, with the latter managed as per the Clavien-Dindo classification.

Data were collected and managed using Microsoft Excel (Microsoft Corp., Redmond, WA), with statistical analysis performed using SPSS Version 22 (IBM Corp., Armonk, NY). Quantitative variables were expressed as mean ± standard deviation or median (interquartile range), and qualitative data were expressed as frequencies and percentages. The association between LEA and independent variables was evaluated using the chi-square test, considering p-values <0.05 as statistically significant.

Ethical aspects

This study was approved by , PGIMER institutional ethics committee (vide letter no. INT/IEC/2022/000518 date March 26, 2022). Informed consent was obtained from all the patients who participated in the study. The study followed the guidelines laid down in the declaration of Helsinki 1964 and as revised later.

## Results

We included 101 patients in the present study. The mean age of the patients was 57.1 years (SD =10.31), with a range of 34 to 80 years (Table 1).

**Table 1:** Description of demographic variables of the patients

Variables	Frequency (n)	Proportion (%)
Gender		
Female	23	22.8
Male	78	77.2
BMI (kg/m^2^)		
Underweight (<18.5)	7	6.9%
Normal (18.5-22.9)	39	38.6%
Overweight (23-24.9)	16	15.8%
Obese grade I (25-29.9)	34	33.7%
Obese grade II (>30)	5	5.0%

**Figure 1:**
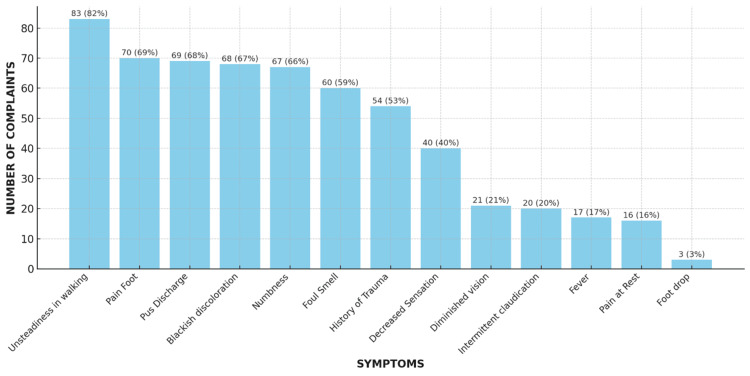
Prevalence of symptoms among patients with diabetic foot complications

The most commonly reported symptom was unsteadiness in walking, accounting for 83 (82%) complaints. This was followed by pain in the foot, reported by 70 (69%) individuals, and pus discharge and blackish discoloration, each with 69 (68%) complaints. Numbness was noted in 67 (66%) cases, while foul smell and a history of trauma were documented in 60 (59%) and 54 (53%) instances, respectively. Decreased sensation accounted for 40 (40%) complaints, and diminished vision was observed in 21 (21%) cases. Intermittent claudication was reported in 20 (20%) cases, fever in 17 (17%) cases, and pain at rest in 16 (16%) cases. Foot drop was the least frequent complaint, recorded in only three (3%) cases. These data highlight the variety of symptoms associated with diabetic foot complications, emphasizing the need for comprehensive management strategies (Figure 1).

The mean duration of ulcer was 63.54 (SD=60.76) days, the mean duration of diabetes mellitus was 11.28 (SD=7.83) years, and the mean duration of anti-diabetic medication intake was 10.07 (SD=7.4) years. Majority of the patients were compliant to antidiabetic medications (54.5%). There were 46 patients who were known cases of hypertension, out of whom 30 patients had controlled and 16 patients had poorly controlled hypertension. The mean duration of hypertension was 8.75 (SD=6.49) years.

While managing the patients, we classified patients into two groups. Group A patients included those who either needed debridement with dressing or those needing minor amputation, whereas group B patients included those who needed major amputation. In our study, debridement was performed in 44 patients, minor amputation was performed in 24 patients, and major amputation was performed in 33 patients. The prevalence of major lower extremity amputation was found to be 32.7% in our study, while that of minor amputation was found to be 23.7% (Table 2).

**Table 2:** Risk factors for amputation *Statistically significant ABI, ankle brachial index; BMI, body mass index; CRP, C-reactive protein; ESR, erythrocyte sedimentation rate; HDL, high-density lipoprotein; LDL, low-density lipoprotein

Predictor	Group A (non-amputation group) (n=69)	Group B (major amputation group) (n=32)	p-Value
Gender (male)	55 (79.7%)	23 (71.9%)	0.382
BMI classification (obesity grade II)	3 (4.3%)	2 (6.3%)	0.413
Wagner's grade ≥ 5	2 (2.9%)	30 (93.8%)	<0.001*
Absent peripheral pulsation	13 (18.8%)	22 (68.8%)	<0.001*
ABI grading (right limb normal)	44 (63.8%)	11 (37.9%)	0.020*
Clinical evidence of infection	36 (52.2%)	31 (96.9%)	<0.001*
Elevated ESR	62 (89.9%)	32 (100%)	0.042
Elevated CRP	66 (95.7%)	32 (100%)	0.031
Elevated cholesterol levels	47 (68.1%)	24 (75%)	0.481
Elevated triglyceride levels	8 (11.6%)	3 (9.4%)	0.739
Low HDL levels	49 (71%)	16 (50%)	0.040
Elevated LDL levels	5 (7.2%)	5 (16.1%)	0.171
poorly controlled HbA1c	61 (89.7%)	26 (78.1%)	0.119
Abnormal urea levels	22 (31.9%)	11 (34.4%)	0.804
Abnormal creatinine levels	25 (36.2%)	9 (28.1%)	0.422
Rutherford classification ≥ 3	12 (17.3%)	11 (35.5%)	0.53

Predominantly, Wagner's grade ≥ 5 emerged as a pivotal predictor, with a pronounced majority in group B presenting with this grade, signifying advanced ulcer severity as a strong indicator of the need for amputation. Additionally, the absence of peripheral pulsation stood out as another critical predictor, markedly prevalent among patients who underwent major amputation. Clinical evidence of infection further underscored its significance, with a considerably higher incidence observed in the major amputation group. Similarly, elevated ESR and CRP levels were significantly associated with the major amputation outcome, highlighting their importance in predicting the necessity for such a drastic measure (Table 2).

## Discussion

DFUs are a significant source of morbidity in individuals with diabetes and are largely preventable. These ulcers can lead to loss of function, infections, hospitalization, LEAs, and even death. The lifetime risk of developing a foot ulcer ranges from 19% to 34%, with this risk increasing as individuals with diabetes live longer and experience more complex medical conditions. With recurrence rates of 65% at three to five years, a lifetime incidence of LEA of 20%, and a five-year mortality rate of 50-70%, the morbidity following initial ulceration is considerable [8].

The present study aimed to estimate the prevalence of major amputations in the lower extremities among patients with DFUs. We enrolled 101 patients, with a mean age of 57.1 years and a male-to-female ratio of 3:1. The mean duration of diabetes was 11.28 years, and the average HbA1c level was 9.1 ± 2.7%, indicating poorly controlled diabetes. A study by Atosona and Larbie reported a male-to-female ratio of 31% to 69%, with a mean age of 53.8 years [9]. Similarly, Wang et al. noted that patients with DFUs were older (mean age 66.96 years), had a longer duration of diabetes (mean 10.30 years), and had higher HbA1c (9.19 ± 2.62%), which aligns closely with the findings of the current study [10].

In this study, the prevalence of major amputations was 32.7%, whereas Elkhider et al. reported a prevalence of 17.1% [11]. The prevalence of amputation among DFU patients in China, as reported by Wang et al., was lower at 5.2% [10]. These differences may be attributed to factors such as genetic variations, differences in diabetes care and management, and the timing of interventions.

Amputation risk varied according to the severity of the diabetic foot condition. Patients with Wagner grades 2-4 had relatively mild foot problems, resulting in a lower amputation rate and a more favorable prognosis. In contrast, patients with Wagner grades above 4 experienced more severe conditions, leading to a higher risk of amputation and greater complications. Wang et al. also analyzed the amputation rates based on Wagner grades and found that during 2004-2013, the amputation rate for DFU patients at Wagner grades 1-2 was 1.95%, with no significant changes over the last decade. However, for patients at grades 3-4, the amputation rate was 19.47%, with significant increases over the same period [9]. Interestingly, body mass index (BMI) was not identified as a risk factor for lower limb amputation or DFUs in the present study. Similar findings were observed by Kim et al., who reported that BMI had no significant impact on LEAs in patients with DFUs [12]. Conversely, Pinzur et al. demonstrated that BMI is associated with LEA, likely due to the effect of body weight on plantar pressure [13].

ABI values lower than 0.8 are indicative of vasculopathy and are associated with increased mortality, morbidity, and hospitalization. The ABI is a reproducible, non-invasive tool used to screen for vasculopathy. In this study, absent peripheral pulsation and an ABI suggestive of moderate arterial disease were significantly associated with major amputation in the lower extremities. Kurniawati et al. reported that 38.2% of patients with an ABI < 0.8 underwent LEA, compared to 6.3% of patients with an ABI ≥ 0.8. ABI < 0.8 was found to be a significant prognostic risk factor for LEA. However, in patients with diabetes, arterial media calcification can falsely elevate the ABI measurement [14]. Sharma et al. also found that medial arterial calcification is linked to increased risks of ulceration, Charcot arthropathy, and mortality in diabetic patients [15].

HbA1c and serum inflammatory markers, such as CRP, have been widely used in diagnosing foot infections in diabetic patients. In this study, LEA was not significantly associated with poorly controlled diabetes (defined as HbA1c > 6.5%) but was found to be significantly linked to elevated CRP levels and the presence of clinical infection. Kurniawati et al. reported that 42.1% of patients with HbA1c > 8.0% underwent limb amputation. Their findings suggested that higher HbA1c is a prognostic risk factor for LEA in DFU patients [13]. Other studies by Sun et al. and Zhou et al. also found a significant association between elevated HbA1c levels and LEA [16,17]. This discrepancy may be due to differences in the HbA1c threshold used to define poorly controlled diabetes across studies.

Dyslipidemia is another major contributor to LEA in patients with DFUs and peripheral vascular disease. However, Rajamani et al. found no significant association between dyslipidemia and LEA in DFU patients, a finding consistent with the present study [18].

Limitations

The study is cross-sectional, meaning it provides a snapshot at a specific point in time. This limits the ability to establish causality between identified risk factors and amputation outcomes. Longitudinal or cohort studies would offer more robust evidence of how risk factors evolve over time and contribute to amputation. The study included 101 patients, which is relatively small. This may limit the statistical power of the findings, particularly in detecting associations for less common risk factors. A larger sample size would improve the reliability and generalizability of the results. The study was conducted at a single institution (PGIMER), which may not fully represent the broader population of patients with DFUs across different regions or healthcare settings. Multi-center studies with diverse patient populations could provide a more generalizable set of results. The exclusion of patients who had received previous endovascular interventions or grafting or those who had cancers or tumors of the lower extremity may limit the applicability of the findings to a specific subset of patients. These exclusions could bias the results, especially regarding the generalizability of the findings to patients with complex medical histories or advanced diabetic complications. Some of the risk factors, such as peripheral pulsation and infection markers (CRP and ESR), are clinical and lab-based assessments, which can vary between practitioners and institutions. The study's reliance on these factors could introduce measurement biases, especially if clinical judgment varies.

## Conclusions

The study conclusively identified several key predictors for major LEA in patients with DFUs, notably advanced ulcer severity (Wagner's grade ≥ 5), absent peripheral pulsation, clinical evidence of infection, and elevated inflammatory markers (ESR and CRP). These findings underscore the critical importance of early and comprehensive clinical assessments, including a detailed examination of ulcer severity, vascular status, and infection markers, in patients presenting with DFUs. In light of these insights, it is recommended that healthcare practitioners implement rigorous screening protocols for these predictors to identify patients at a high risk for amputation. Early intervention strategies, including aggressive management of infections, optimization of vascular health, and meticulous wound care, should be prioritized to mitigate the risk of major amputation and improve outcomes for this vulnerable patient population.

## References

[1] Lavery LA, Armstrong DG, Wunderlich RP, Tredwell J, Boulton AJ (2003). Diabetic foot syndrome: evaluating the prevalence and incidence of foot pathology in Mexican Americans and non-Hispanic whites from a diabetes disease management cohort. Diabetes Care.

[2] Shatnawi NJ, Al-Zoubi NA, Hawamdeh HM, Khader YS, Garaibeh K, Heis HA (2018). Predictors of major lower limb amputation in type 2 diabetic patients referred for hospital care with diabetic foot syndrome. Diabetes Metab Syndr Obes.

[3] Acar E, Kacıra BK (2017). Predictors of lower extremity amputation and reamputation associated with the diabetic foot. J Foot Ankle Surg.

[4] Ugwu E, Adeleye O, Gezawa I, Okpe I, Enamino M, Ezeani I (2019). Predictors of lower extremity amputation in patients with diabetic foot ulcer: findings from MEDFUN, a multi-center observational study. J Foot Ankle Res.

[5] Umashankar GT, S AKM, Shahid M (2019). Predictors of lower extremity amputation in patients with diabetic foot ulcer. Int Surg J.

[6] Lin C, Liu J, Sun H (2020). Risk factors for lower extremity amputation in patients with diabetic foot ulcers: a meta-analysis. PLoS One.

[7] Perng CK, Chou HY, Chiu YJ (2021). Identifying major predictors of lower-extremity amputation in patients with diabetic foot ulcers. J Chin Med Assoc.

[8] McDermott K, Fang M, Boulton AJ, Selvin E, Hicks CW (2023). Etiology, epidemiology, and disparities in the burden of diabetic foot ulcers. Diabetes Care.

[9] Atosona A, Larbie C (2019). Prevalence and determinants of diabetic foot ulcers and lower extremity amputations in three selected tertiary hospitals in Ghana. J Diabetes Res.

[10] Wang C, Mai L, Yang C (2016). Reducing major lower extremity amputations after the introduction of a multidisciplinary team in patient with diabetes foot ulcer. BMC Endocr Disord.

[11] Elkhider AT, Almobark AO, Badi S (2021). Risk factors associated with lower extremity amputation in Sudanese individuals with diabetes: the need for improvement in primary health care system. J Family Med Prim Care.

[12] 1] Kim JK, Jung YR, Kim KT, Shin CS, Lee KB (2017). A Report on Diabetic Foot and Amputation from the Korean Health Insurance Review & Assessment Service Data. J Korean Foot Ankle Soc.

[13] Pinzur M, Freeland R, Juknelis D (2005). The association between body mass index and foot disorders in diabetic patients. Foot Ankle Int.

[14] Kurniawati A, Ismiarto YD, Hsu IL (2019). Prognostic factors for lower extremity amputation in diabetic foot ulcer patients. J Acute Med.

[15] Sharma A, Scammell BE, Fairbairn KJ, Seagrave MJ, Game FL, Jeffcoate WJ (2010). Prevalence of calcification in the pedal arteries in diabetes complicated by foot disease. Diabetes Care.

[16] Sun JH, Tsai JS, Huang CH (2012). Risk factors for lower extremity amputation in diabetic foot disease categorized by Wagner classification. Diabetes Res Clin Pract.

[17] Zhou ZY, Liu YK, Chen HL, Yang HL, Liu F (2015). HbA1c and lower extremity amputation risk in patients with diabetes: a meta-analysis. Int J Low Extrem Wounds.

[18] Rajamani K, Li LP, Kesaniemi YA (2009). Abstract 1040: lower-limb amputation in patients with type 2 diabetes mellitus: factors predicting risk in the FIELD study. Circulation.

